# State-of-the-art literature review methodology: A six-step approach for knowledge synthesis

**DOI:** 10.1007/s40037-022-00725-9

**Published:** 2022-09-05

**Authors:** Erin S. Barry, Jerusalem Merkebu, Lara Varpio

**Affiliations:** 1 Department of Anesthesiology, F. Edward Hébert School of Medicine, Uniformed Services University, Bethesda, MD USA; 2 School of Health Professions Education (SHE), Maastricht University, Maastricht, The Netherlands; 3 Department of Medicine, F. Edward Hébert School of Medicine, Uniformed Services University, Bethesda, MD USA

**Keywords:** State-of-the-art literature review, Literature review, Literature review methodology

## Abstract

**Introduction:**

Researchers and practitioners rely on literature reviews to synthesize large bodies of knowledge. Many types of literature reviews have been developed, each targeting a specific purpose. However, these syntheses are hampered if the review type’s paradigmatic roots, methods, and markers of rigor are only vaguely understood. One literature review type whose methodology has yet to be elucidated is the state-of-the-art (SotA) review. If medical educators are to harness SotA reviews to generate knowledge syntheses, we must understand and articulate the paradigmatic roots of, and methods for, conducting SotA reviews.

**Methods:**

We reviewed 940 articles published between 2014–2021 labeled as SotA reviews. We (a) identified all SotA methods-related resources, (b) examined the foundational principles and techniques underpinning the reviews, and (c) combined our findings to inductively analyze and articulate the philosophical foundations, process steps, and markers of rigor.

**Results:**

In the 940 articles reviewed, nearly all manuscripts (98%) lacked citations for how to conduct a SotA review. The term “state of the art” was used in 4 different ways. Analysis revealed that SotA articles are grounded in relativism and subjectivism.

**Discussion:**

This article provides a 6-step approach for conducting SotA reviews. SotA reviews offer an interpretive synthesis that describes: *This is where we are now. This is how we got here. This is where we could be going.* This chronologically rooted narrative synthesis provides a methodology for reviewing large bodies of literature to explore why and how our current knowledge has developed and to offer new research directions.

**Supplementary Information:**

The online version of this article (10.1007/s40037-022-00725-9) contains supplementary material, which is available to authorized users.

## Background

Literature reviews play a foundational role in scientific research; they support knowledge advancement by collecting, describing, analyzing, and integrating large bodies of information and data [1, 2]. Indeed, as Snyder [3] argues, all scientific disciplines require literature reviews grounded in a methodology that is accurate and clearly reported. Many types of literature reviews have been developed, each with a unique purpose, distinct methods, and distinguishing characteristics of quality and rigor [4, 5].

Each review type offers valuable insights if rigorously conducted [3, 6]. Problematically, this is not consistently the case, and the consequences can be dire. Medical education’s policy makers and institutional leaders rely on knowledge syntheses to inform decision making [7]. Medical education curricula are shaped by these syntheses. Our accreditation standards are informed by these integrations. Our patient care is guided by these knowledge consolidations [8]. Clearly, it is important for knowledge syntheses to be held to the highest standards of rigor. And yet, that standard is not always maintained. Sometimes scholars fail to meet the review’s specified standards of rigor; other times the markers of rigor have never been explicitly articulated. While we can do little about the former, we can address the latter. One popular literature review type whose methodology has yet to be fully described, vetted, and justified is the state-of-the-art (SotA) review.

While many types of literature reviews amalgamate bodies of literature, SotA reviews offer something unique. By looking across the historical development of a body of knowledge, SotA reviews delves into questions like: Why did our knowledge evolve in this way? What other directions might our investigations have taken? What turning points in our thinking should we revisit to gain new insights? A SotA review—a form of narrative knowledge synthesis [5, 9]—acknowledges that history reflects a series of decisions and then asks what different decisions might have been made.

SotA reviews are frequently used in many fields including the biomedical sciences [10, 11], medicine [12–14], and engineering [15, 16]. However, SotA reviews are rarely seen in medical education; indeed, a bibliometrics analysis of literature reviews published in 14 core medical education journals between 1999 and 2019 reported only 5 SotA reviews out of the 963 knowledge syntheses identified [17]. This is not to say that SotA reviews are absent; we suggest that they are often unlabeled. For instance, Schuwirth and van der Vleuten’s article “A history of assessment in medical education” [14] offers a temporally organized overview of the field’s evolving thinking about assessment. Similarly, McGaghie et al. published a chronologically structured review of simulation-based medical education research that “reviews and critically evaluates historical and contemporary research on simulation-based medical education” [18, p. 50]. SotA reviews certainly have a place in medical education, even if that place is not explicitly signaled.

This lack of labeling is problematic since it conceals the purpose of, and work involved in, the SotA review synthesis. In a SotA review, the author(s) collects and analyzes the historical development of a field’s knowledge about a phenomenon, deconstructs how that understanding evolved, questions why it unfolded in specific ways, and posits new directions for research. Senior medical education scholars use SotA reviews to share their insights based on decades of work on a topic [14, 18]; their junior counterparts use them to critique that history and propose new directions [19]. And yet, SotA reviews are generally not explicitly signaled in medical education. We suggest that at least two factors contribute to this problem. First, it may be that medical education scholars have yet to fully grasp the unique contributions SotA reviews provide. Second, the methodology and methods of SotA reviews are poorly reported making this form of knowledge synthesis appear to lack rigor. Both factors are rooted in the same foundational problem: insufficient clarity about SotA reviews. In this study, we describe SotA review methodology so that medical educators can explicitly use this form of knowledge synthesis to further advance the field.

## Methods

**Fig. 1:**
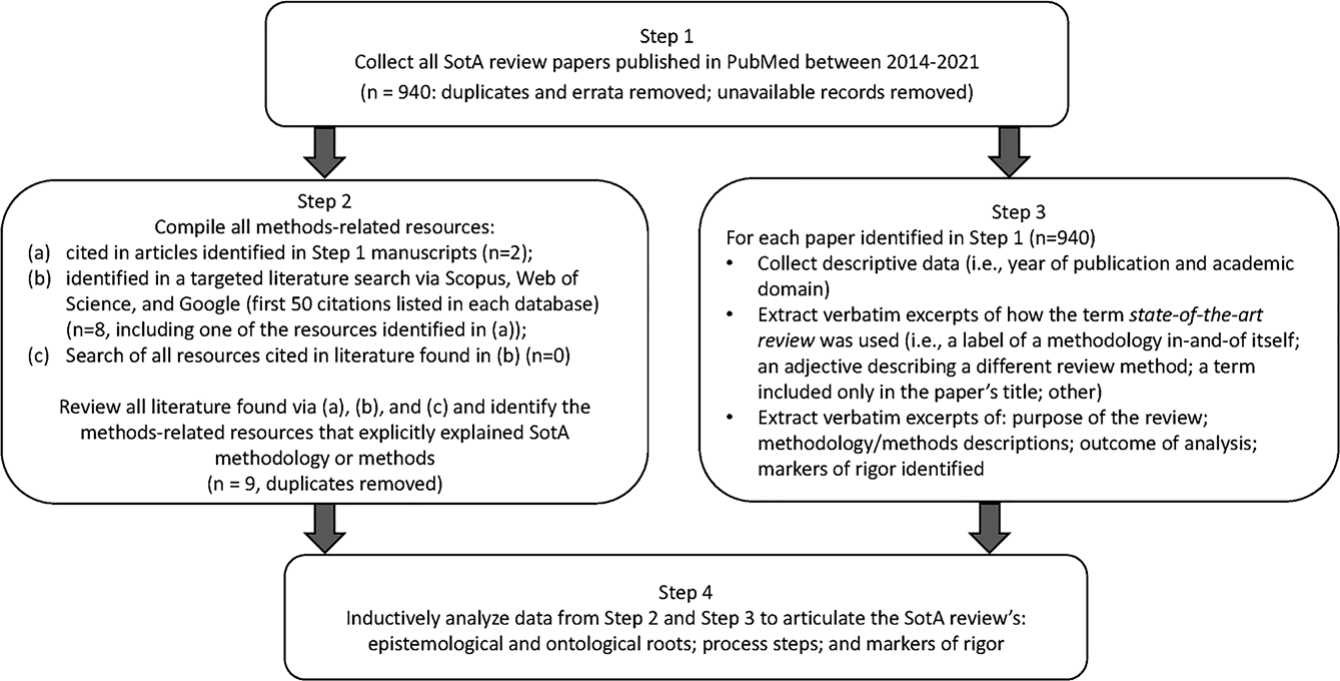
Four-step research design process used for developing a State-of-the-Art literature review methodology

We developed a four-step research design to meet this goal, illustrated in Fig. 1.

### Step 1: Collect SotA articles

To build our initial corpus of articles reporting SotA reviews, we searched PubMed using the strategy (″state of the art review″[ti] OR ″state of the art review*″) and limiting our search to English articles published between 2014 and 2021. We strategically focused on PubMed, which includes MEDLINE, and is considered the National Library of Medicine’s premier database of biomedical literature and indexes health professions education and practice literature [20]. We limited our search to 2014–2021 to capture modern use of SotA reviews. Of the 960 articles identified, nine were excluded because they were duplicates, erratum, or corrigendum records; full text copies were unavailable for 11 records. All articles identified (*n* = 940) constituted the corpus for analysis.

### Step 2: Compile all methods-related resources

EB, JM, or LV independently reviewed the 940 full-text articles to identify all references to resources that explained, informed, described, or otherwise supported the methods used for conducting the SotA review. Articles that met our criteria were obtained for analysis.

To ensure comprehensive retrieval, we also searched Scopus and Web of Science. Additionally, to find resources not indexed by these academic databases, we searched Google (see Electronic Supplementary Material [ESM] for the search strategies used for each database). EB also reviewed the first 50 items retrieved from each search looking for additional relevant resources. None were identified. Via these strategies, nine articles were identified and added to the collection of methods-related resources for analysis.

### Step 3: Extract data for analysis

In Step 3, we extracted three kinds of information from the 940 articles papers identified in Step 1. First, descriptive data on each article were compiled (i.e., year of publication and the academic domain targeted by the journal). Second, each article was examined and excerpts collected about how the term *state-of-the-art review* was used (i.e., as a label for a methodology in-and-of itself; as an adjective qualifying another type of literature review; as a term included in the paper’s title only; or in some other way). Finally, we extracted excerpts describing: the purposes and/or aims of the SotA review; the methodology informing and methods processes used to carry out the SotA review; outcomes of analyses; and markers of rigor for the SotA review.

Two researchers (EB and JM) coded 69 articles and an interrater reliability of 94.2% was achieved. Any discrepancies were discussed. Given the high interrater reliability, the two authors split the remaining articles and coded independently.

### Step 4: Construct the SotA review methodology

The methods-related resources identified in Step 2 and the data extractions from Step 3 were inductively analyzed by LV and EB to identify statements and research processes that revealed the ontology (i.e., the nature of reality that was reflected) and the epistemology (i.e., the nature of knowledge) underpinning the descriptions of the reviews. These authors studied these data to determine if the synthesis adhered to an objectivist or a subjectivist orientation, and to synthesize the purposes realized in these papers.

To confirm these interpretations, LV and EB compared their ontology, epistemology, and purpose determinations against two expectations commonly required of objectivist synthesis methods (e.g., systematic reviews): an exhaustive search strategy and an appraisal of the quality of the research data. These expectations were considered indicators of a realist ontology and objectivist epistemology [21] (i.e., that a single correct understanding of the topic can be sought through objective data collection {e.g., systematic reviews [22]}). Conversely, the inverse of these expectations were considered indicators of a relativist ontology and subjectivist epistemology [21] (i.e., that no single correct understanding of the topic is available; there are multiple valid understandings that can be generated and so a subjective interpretation of the literature is sought {e.g., narrative reviews [9]}).

Once these interpretations were confirmed, LV and EB reviewed and consolidated the methods steps described in these data. Markers of rigor were then developed that aligned with the ontology, epistemology, and methods of SotA reviews.

## Results

Of the 940 articles identified in Step 1, 98% (*n* = 923) lacked citations or other references to resources that explained, informed, or otherwise supported the SotA review process. Of the 17 articles that included supporting information, 16 cited Grant and Booth’s description [4] consisting of five sentences describing the overall purpose of SotA reviews, three sentences noting perceived strengths, and four sentences articulating perceived weaknesses. This resource provides no guidance on how to conduct a SotA review methodology nor markers of rigor. The one article not referencing Grant and Booth used “an adapted comparative effectiveness research search strategy that was adapted by a health sciences librarian” [23, p. 381]. One website citation was listed in support of this strategy; however, the page was no longer available in summer 2021. We determined that the corpus was uninformed by a cardinal resource or a publicly available methodology description.

In Step 2 we identified nine resources [4, 5, 24–28]; none described the methodology and/or processes of carrying out SotA reviews. Nor did they offer explicit descriptions of the ontology or epistemology underpinning SotA reviews. Instead, these resources provided short overview statements (none longer than one paragraph) about the review type [4, 5, 24–28]. Thus, we determined that, to date, there are no available methodology papers describing how to conduct a SotA review.

**Fig. 2:**
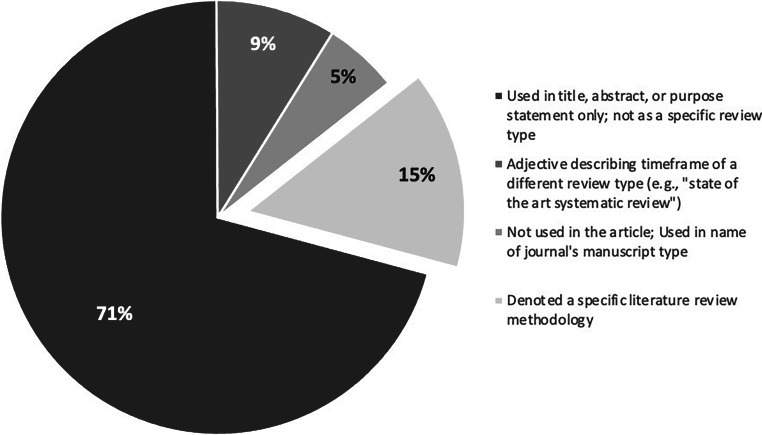
Four ways the term “state of the art” is used in the corpus and how frequently each is used

Step 3 revealed that “state of the art” was used in 4 different ways across the 940 articles (see Fig. 2 for the frequency with which each was used). In 71% (*n* = 665 articles), the phrase was used only in the title, abstract, and/or purpose statement of the article; the phrase did not appear elsewhere in the paper and no SotA methodology was discussed. Nine percent (*n* = 84) used the phrase as an adjective to qualify another literature review type and so relied entirely on the methodology of a different knowledge synthesis approach (e.g., “a state of the art systematic review [29]”). In 5% (*n* = 52) of the articles, the phrase was not used anywhere within the article; instead, “state of the art” was the type of article within a journal. In the remaining 15% (*n* = 139), the phrase denoted a specific methodology (see ESM for all methodology articles). Via Step 4’s inductive analysis, the following foundational principles of SotA reviews were developed: (1) the ontology, (2) epistemology, and (3) purpose of SotA reviews.

### Ontology of SotA reviews: Relativism

SotA reviews rest on four propositions:

1. The literature addressing a phenomenon offers multiple perspectives on that topic (i.e., different groups of researchers may hold differing opinions and/or interpretations of data about a phenomenon).
2. The reality of the phenomenon itself cannot be completely perceived or understood (i.e., due to limitations [e.g., the capabilities of current technologies, a research team’s disciplinary orientation] we can only perceive a limited part of the phenomenon).
3. The reality of the phenomenon is a subjective and inter-subjective construction (i.e., what we understand about a phenomenon is built by individuals and so their individual subjectivities shape that understanding).
4. The context in which the review was conducted informs the review (e.g., a SotA review of literature about gender identity and sexual function will be synthesized differently by researchers in the domain of gender studies than by scholars working in sex reassignment surgery).

As these propositions suggest, SotA scholars bring their experiences, expectations, research purposes, and social (including academic) orientations to bear on the synthesis work. In other words, a SotA review synthesizes the literature based on a specific orientation to the topic being addressed. For instance, a SotA review written by senior scholars who are experts in the field of medical education may reflect on the turning points that have shaped the way our field has evolved the modern practices of learner assessment, noting how the nature of the problem of assessment has moved: it was first a measurement problem, then a problem that embraced human judgment but needed assessment expertise, and now a whole system problem that is to be addressed from an integrated—not a reductionist—perspective [12]. However, if other scholars were to examine this same history from a technological orientation, learner assessment could be framed as historically constricted by the media available through which to conduct assessment, pointing to how artificial intelligence is laying the foundation for the next wave of assessment in medical education [30].

Given these foundational propositions, SotA reviews are steeped in a relativist ontology—i.e., reality is socially and experientially informed and constructed, and so no single objective truth exists. Researchers’ interpretations reflect their conceptualization of the literature—a conceptualization that could change over time and that could conflict with the understandings of others.

### Epistemology of SotA reviews: Subjectivism

SotA reviews embrace subjectivism. The knowledge generated through the review is value-dependent, growing out of the subjective interpretations of the researcher(s) who conducted the synthesis. The SotA review generates an interpretation of the data that is informed by the expertise, experiences, and social contexts of the researcher(s). Furthermore, the knowledge developed through SotA reviews is shaped by the historical point in time when the review was conducted. SotA reviews are thus steeped in the perspective that knowledge is shaped by individuals and their community, and is a synthesis that will change over time.

### Purpose of SotA reviews

SotA reviews create a subjectively informed summary of modern thinking about a topic. As a chronologically ordered synthesis, SotA reviews describe the history of turning points in researchers’ understanding of a phenomenon to contextualize a description of modern scientific thinking on the topic. The review presents an argument about how the literature *could* be interpreted; it is not a definitive statement about how the literature *should *or *must *be interpreted. A SotA review explores: the pivotal points shaping the historical development of a topic, the factors that informed those changes in understanding, and the ways of thinking about and studying the topic that could inform the generation of further insights. In other words, the purpose of SotA reviews is to create a three-part argument: *This is where we are now in our understanding of this topic. This is how we got here. This is where we could go next.*

### The SotA methodology

Based on study findings and analyses, we constructed a six-stage SotA review methodology. This six-stage approach is summarized and guiding questions are offered in Tab. 1.

**Table 1:** The six-stage approach to conducting a State-of-the-Art review

Stage	Guiding Questions	Examples of thoughts as related to interprofessional education (IPE)
*Stage 1:* Determine initial research question and field of inquiry	– What is (are) the research question(s) to be addressed? – What field of knowledge and/or practice will the search address?	How has thinking about IPE evolved? What are the modern ways of thinking about and doing IPE?
*Stage 2:* Determine timeframe	– Engage in a broad-scope overview around the topic to be addressed – What historical markers help demarcate the timeframe of now? – What timeframe can be justified to mark the beginning of the review?	In 2010, the World Health Organization defined IPE [31]. This is a sentinel moment that could be considered the start of modern, state-of-the art thinking on IPE
*Stage 3:* Finalize research question(s) to reflect timeframe	– Do the broad-scope overview and historical markers change your research question(s)? – Does this information require you to adjust your research question(s)?	What is the state-of-the-art way of conceptualizing and realizing IPE?
*Stage 4:* Develop search strategy to find relevant manuscripts	– How far back on your timeframe do you need to go to report “this is how we got here”? – How could a librarian consultation enhance your search strategy?	Given the Stage 2 finding, the search strategy will focus on (i) identifying changes in conceptualizing and realizing IPE pre-2010; and (ii) describing how IPE has been conceptualized and realized post-2010
*Stage 5:* Analyses	– Read the articles to become familiar with the literature – What are the similarities across articles? – What are the assumptions underpinning changes in understanding the topic over time? – What are the gaps and assumptions in current knowledge? – Which articles support/contradict your thinking? – Does the literature reflect the premise you set out to study?	Analysis will identify pivotal moments in the IPE literature, focusing on what came before the 2010 definition, what came after 2010, and what future IPE researchers might consider
	This is how we got here & This is where we are now: – What is the history that gave rise to the modern way of thinking? – Which theories have shaped insights and understandings?	
	This is where we could be going – What are the future directions of research? – Do certain authors dominate the literature? – Are there any marginalized points of view that should be considered?	
*Stage 6:* Reflexivity	– Provide a reflexivity description	A robust reflexivity description is provided to explain how researcher subjectivities shaped interpretations of the IPE literature

#### Stage 1: Determine initial research question and field of inquiry

In Stage 1, the researcher(s) creates an initial description of the topic to be summarized and so must determine what field of knowledge (and/or practice) the search will address. Knowledge developed through the SotA review process is shaped by the context informing it; thus, knowing the domain in which the review will be conducted is part of the review’s foundational work.

#### Stage 2: Determine timeframe

This stage involves determining the period of time that will be defined as SotA for the topic being summarized. The researcher(s) should engage in a broad-scope overview of the literature, reading across the range of literature available to develop insights into the historical development of knowledge on the topic, including the turning points that shape the current ways of thinking about a topic. Understanding the full body of literature is required to decide the dates or events that demarcate the timeframe of *now* in the first of the SotA’s three-part argument: *where we are now*. Stage 2 is complete when the researcher(s) can explicitly justify why a specific year or event is the right moment to mark the beginning of state-of-the-art thinking about the topic being summarized.

#### Stage 3: Finalize research question(s) to reflect timeframe

Based on the insights developed in Stage 2, the researcher(s) will likely need to revise their initial description of the topic to be summarized. The formal research question(s) framing the SotA review are finalized in Stage 3. The revised description of the topic, the research question(s), and the justification for the timeline start year must be reported in the review article. These are markers of rigor and prerequisites for moving to Stage 4.

#### Stage 4: Develop search strategy to find relevant articles

In Stage 4, the researcher(s) develops a search strategy to identify the literature that will be included in the SotA review. The researcher(s) needs to determine which literature databases contain articles from the domain of interest. Because the review describes *how we got here*, the review must include literature that predates the state-of-the-art timeframe, determined in Stage 2, to offer this historical perspective.

Developing the search strategy will be an iterative process of testing and revising the search strategy to enable the researcher(s) to capture the breadth of literature required to meet the SotA review purposes. A librarian should be consulted since their expertise can expedite the search processes and ensure that relevant resources are identified. The search strategy must be reported (e.g., in the manuscript itself or in a supplemental file) so that others may replicate the process if they so choose (e.g., to construct a different SotA review [and possible different interpretations] of the same literature). This too is a marker of rigor for SotA reviews: the search strategies informing the identification of literature must be reported.

#### Stage 5: Analyses

The literature analysis undertaken will reflect the subjective insights of the researcher(s); however, the foundational premises of inductive research should inform the analysis process. Therefore, the researcher(s) should begin by reading the articles in the corpus to become familiar with the literature. This familiarization work includes: noting similarities across articles, observing ways-of-thinking that have shaped current understandings of the topic, remarking on assumptions underpinning changes in understandings, identifying important decision points in the evolution of understanding, and taking notice of gaps and assumptions in current knowledge.

The researcher(s) can then generate premises for the state-of-the-art understanding of the history that gave rise to modern thinking, of the current body of knowledge, and of potential future directions for research. In this stage of the analysis, the researcher(s) should document the articles that support or contradict their premises, noting any collections of authors or schools of thinking that have dominated the literature, searching for marginalized points of view, and studying the factors that contributed to the dominance of particular ways of thinking. The researcher(s) should also observe historical decision points that could be revisited. Theory can be incorporated at this stage to help shape insights and understandings. It should be highlighted that not all corpus articles will be used in the SotA review; instead, the researcher(s) will sample across the corpus to construct a timeline that represents the seminal moments of the historical development of knowledge.

Next, the researcher(s) should verify the thoroughness and strength of their interpretations. To do this, the researcher(s) can select different articles included in the corpus and examine if those articles reflect the premises the researcher(s) set out. The researcher(s) may also seek out contradictory interpretations in the literature to be sure their summary refutes these positions. The goal of this verification work is not to engage in a triangulation process to ensure objectivity; instead, this process helps the researcher(s) ensure the interpretations made in the SotA review represent the articles being synthesized and respond to the interpretations offered by others. This is another marker of rigor for SotA reviews: the authors should engage in and report how they considered and accounted for differing interpretations of the literature, and how they verified the thoroughness of their interpretations.

#### Stage 6: Reflexivity

Given the relativist subjectivism of a SotA review, it is important that the manuscript offer insights into the subjectivity of the researcher(s). This reflexivity description should articulate how the subjectivity of the researcher(s) informed interpretations of the data. These reflections will also influence the suggested directions offered in the last part of the SotA three-part argument: *where we could go next.* This is the last marker of rigor for SotA reviews: researcher reflexivity must be considered and reported.

## Discussion

SotA reviews have much to offer our field since they provide information on the historical progression of medical education’s understanding of a topic, the turning points that guided that understanding, and the potential next directions for future research. Those future directions may question the soundness of turning points and prior decisions, and thereby offer new paths of investigation. Since we were unable to find a description of the SotA review methodology, we inductively developed a description of the methodology—including its paradigmatic roots, the processes to be followed, and the markers of rigor—so that scholars can harness the unique affordances of this type of knowledge synthesis.

Given their chronology- and turning point-based orientation, SotA reviews are inherently different from other types of knowledge synthesis. For example, systematic reviews focus on specific research questions that are narrow in scope [32, 33]; in contrast, SotA reviews present a broader historical overview of knowledge development and the decisions that gave rise to our modern understandings. Scoping reviews focus on mapping the present state of knowledge about a phenomenon including, for example, the data that are currently available, the nature of that data, and the gaps in knowledge [34, 35]; conversely, SotA reviews offer interpretations of the historical progression of knowledge relating to a phenomenon centered on significant shifts that occurred during that history. SotA reviews focus on the turning points in the history of knowledge development to suggest how different decisions could give rise to new insights. Critical reviews draw on literature outside of the domain of focus to see if external literature can offer new ways of thinking about the phenomenon of interest (e.g., drawing on insights from insects’ swarm intelligence to better understand healthcare team adaptation [36]). SotA reviews focus on one domain’s body of literature to construct a timeline of knowledge development, demarcating where we are now, demonstrating how this understanding came to be via different turning points, and offering new research directions. Certainly, SotA reviews offer a unique kind of knowledge synthesis.

Our six-stage process for conducting these reviews reflects the subjectivist relativism that underpins the methodology. It aligns with the requirements proposed by others [24–27], what has been written about SotA reviews [4, 5], and the current body of published SotA reviews. In contrast to existing guidance [4, 5, 20–23], our description offers a detailed reporting of the ontology, epistemology, and methodology processes for conducting the SotA review.

This explicit methodology description is essential since many academic journals list SotA reviews as an accepted type of literature review. For instance, *Educational Research Review *[24], the American Academy of Pediatrics [25], and *Thorax* all lists SotA reviews as one of the types of knowledge syntheses they accept [27]. However, while SotA reviews are valued by academia, guidelines or specific methodology descriptions for researchers to follow when conducting this type of knowledge synthesis are conspicuously absent. If academics in general, and medical education more specifically, are to take advantage of the insights that SotA reviews can offer, we need to rigorously engage in this synthesis work; to do that, we need clear descriptions of the methodology underpinning this review. This article offers such a description. We hope that more medical educators will conduct SotA reviews to generate insights that will contribute to further advancing our field’s research and scholarship.

## Supplementary Information

For information regarding the search strategy to develop the corpus and search strategy for confirming capture of any available State of the Art review methodology descriptions. Additionally, a list of the methodology articles found through the search strategy/corpus is included
