# Supplementary material for: State-of-the-art literature review methodology: A six-step approach for knowledge synthesis
Source: Perspect Med Educ. 2022 Sep 5;11(5):281–8. doi: 10.1007/s40037-022-00725-9 (PMC9582072; doi:10.1007/s40037-022-00725-9)
Supplement: Supplementary file 1 — For information regarding the search strategy to develop the corpus and search strategy for confirming capture of any available State of the Art review methodology descriptions. Additionally, a list of the methodology articles found through the search strategy/corpus is included [file 40037_2022_725_MOESM1_ESM.docx]

**Electronic Supplementary Material (ESM)**

Search strategy for developing the corpus and search strategy for confirming capture of any available State of the Art review methodology descriptions

| *PubMed*  ("state of the art review"[ti] OR "state of the art review*") AND english[lang] AND ("2014/01/01"[PDAT]: "3000/12/31"[PDAT])  Search conducted on 8/10/2021, updated on 8/10/2021.  *Scopus*  ("state of the art review"[ti] OR "state of the art review*") AND english[lang]  Search conducted on 10/1/2021.  *Web of Science*  ("state of the art review"[ti] OR "state of the art review*") AND english[lang]  Search conducted on 10/1/2021.  *Google*  “state of the art review”  Search conducted on 10/1/2021. |
| --- |

Scopus, Web of Science, and Google results were searched based on relevance rankings.

**State-of-the-Art methodology articles (N=139)**

1. Abou Ziki MD, Mani A. Wnt signaling, a novel pathway regulating blood pressure? State of the art review. Atherosclerosis. 2017;262:171-8.
2. Alexis SL, Malik AH, George I, Hahn RT, Khalique OK, Seetharam K, et al. Infective Endocarditis After Surgical and Transcatheter Aortic Valve Replacement: A State of the Art Review. J Am Heart Assoc. 2020;9(16):e017347.
3. Alio L, Angioni S, Arena S, Bartiromo L, Bergamini V, Berlanda N, et al. Endometriosis: seeking optimal management in women approaching menopause. Climacteric. 2019;22(4):329-38.
4. Andreasson AS, Dark JH, Fisher AJ. Ex vivo lung perfusion in clinical lung transplantation--state of the art. Eur J Cardiothorac Surg. 2014;46(5):779-88.
5. Arena A, Prete F, Rambaldi E, Bignozzi MC, Monaco C, Di Fiore A, et al. Nanostructured Zirconia-Based Ceramics and Composites in Dentistry: A State-of-the-Art Review. Nanomaterials (Basel, Switzerland). 2019;9(10):1393.
6. Arkenbout EA, Henselmans PW, Jelínek F, Breedveld P. A state of the art review and categorization of multi-branched instruments for NOTES and SILS. Surg Endosc. 2015;29(6):1281-96.
7. Avitabile E, Bedognetti D, Ciofani G, Bianco A, Delogu LG. How can nanotechnology help the fight against breast cancer? Nanoscale. 2018;10(25):11719-31.
8. Badillo-Suárez PA, Rodríguez-Cruz M, Nieves-Morales X. Impact of Metabolic Hormones Secreted in Human Breast Milk on Nutritional Programming in Childhood Obesity. J Mammary Gland Biol Neoplasia. 2017;22(3):171-91.
9. Banchhor SK, Londhe ND, Araki T, Saba L, Radeva P, Khanna NN, et al. Calcium detection, its quantification, and grayscale morphology-based risk stratification using machine learning in multimodality big data coronary and carotid scans: A review. Comput Biol Med. 2018;101:184-98.
10. Barlow CA, Sahmel J, Paustenbach DJ, Henshaw JL. History of knowledge and evolution of occupational health and regulatory aspects of asbestos exposure science: 1900-1975. Crit Rev Toxicol. 2017;47(4):286-316.
11. Baskin KM, Mermel LA, Saad TF, Journeycake JM, Schaefer CM, Modi BP, et al. Evidence-Based Strategies and Recommendations for Preservation of Central Venous Access in Children. JPEN J Parenter Enteral Nutr. 2019;43(5):591-614.
12. Bates ER, Tamis-Holland JE, Bittl JA, O'Gara PT, Levine GN. PCI Strategies in Patients With ST-Segment Elevation Myocardial Infarction and Multivessel Coronary Artery Disease. J Am Coll Cardiol. 2016;68(10):1066-81.
13. Bell EB, Cohen ER, Sargi Z, Leibowitz J. Free tissue reconstruction of the anterior skull base: A review. World J Otorhinolaryngol Head Neck Surg. 2020;6(2):132-6.
14. Bikdeli B, Madhavan MV, Jimenez D, Chuich T, Dreyfus I, Driggin E, et al. COVID-19 and Thrombotic or Thromboembolic Disease: Implications for Prevention, Antithrombotic Therapy, and Follow-Up: JACC State-of-the-Art Review. J Am Coll Cardiol. 2020;75(23):2950-73.
15. Bonini M, Di Paolo M, Bagnasco D, Baiardini I, Braido F, Caminati M, et al. Minimal clinically important difference for asthma endpoints: an expert consensus report. Eur Respir Rev. 2020;29(156).
16. Bowe SN, Schmalbach CE, Laury AM. The State of the Otolaryngology Match: A Review of Applicant Trends, "Impossible" Qualifications, and Implications. Otolaryngol Head Neck Surg. 2017;156(6):985-90.
17. Cannata A, Cantoni S, Sciortino A, Bruschi G, Russo CF. Mechanical Hemolysis Complicating Transcatheter Interventions for Valvular Heart Disease: JACC State-of-the-Art Review. J Am Coll Cardiol. 2021;77(18):2323-34.
18. Cant RP, Cooper SJ. The value of simulation-based learning in pre-licensure nurse education: A state-of-the-art review and meta-analysis. Nurse education in practice. 2017;27:45-62.
19. Carbone S, Dixon DL, Buckley LF, Abbate A. Glucose-Lowering Therapies for Cardiovascular Risk Reduction in Type 2 Diabetes Mellitus: State-of-the-Art Review. Mayo Clinic proceedings. 2018;93(11):1629-47.
20. Casanueva A, Burgstall A, Kotlarski S, Messeri A, Morabito M, Flouris AD, et al. Overview of Existing Heat-Health Warning Systems in Europe. Int J Environ Res Public Health. 2019;16(15).
21. Chandra SS, Bran Lorenzana M, Liu X, Liu S, Bollmann S, Crozier S. Deep learning in magnetic resonance image reconstruction. J Med Imaging Radiat Oncol. 2021.
22. Chiang T, Pepper V, Best C, Onwuka E, Breuer CK. Clinical Translation of Tissue Engineered Trachea Grafts. Ann Otol Rhinol Laryngol. 2016;125(11):873-85.
23. Chong WH, Saha BK, Austin A, Chopra A. The Significance of Subpleural Sparing in CT Chest: A State-of-the-Art Review. Am J Med Sci. 2021;361(4):427-35.
24. Chong WH, Saha BK, Conuel E, Chopra A. The incidence of pleural effusion in COVID-19 pneumonia: State-of-the-art review. Heart Lung. 2021;50(4):481-90.
25. Corkery JM, Streete P, Claridge H, Goodair C, Papanti D, Orsolini L, et al. Characteristics of deaths associated with kratom use. J Psychopharmacol. 2019;33(9):1102-23.
26. Cramer JD, Barnett ML, Anne S, Bateman BT, Rosenfeld RM, Tunkel DE, et al. Nonopioid, Multimodal Analgesia as First-line Therapy After Otolaryngology Operations: Primer on Nonsteroidal Anti-inflammatory Drugs (NSAIDs). Otolaryngol Head Neck Surg. 2021;164(4):712-9.
27. Cramer JD, Wisler B, Gouveia CJ. Opioid Stewardship in Otolaryngology: State of the Art Review. Otolaryngol Head Neck Surg. 2018;158(5):817-27.
28. Cross JH, Prentice AM, Cerami C. Hepcidin, Serum Iron, and Transferrin Saturation in Full-Term and Premature Infants during the First Month of Life: A State-of-the-Art Review of Existing Evidence in Humans. Curr Dev Nutr. 2020;4(8):nzaa104.
29. Cullen PP, Tsui SS, Caplice NM, Hinchion JA. A state-of-the-art review of the current role of cardioprotective techniques in cardiac transplantation. Interact Cardiovasc Thorac Surg. 2021;32(5):683-94.
30. Dack D, Ban S. To what extent does the use of a coaching-based style of student supervision in clinical practice impact experiences of placements for staff and students: A state-of-the-art literature review. Nurse Educ Today. 2021;103:104941.
31. Darnton-Hill I. Public Health Aspects in the Prevention and Control of Vitamin Deficiencies. Curr Dev Nutr. 2019;3(9):nzz075.
32. De Lisi S, Giovannini M. Endoscopic ultrasonography: Transition towards the future of gastro-intestinal diseases. World J Gastroenterol. 2016;22(5):1779-86.
33. De Stefani A, Bruno G, Preo G, Gracco A. Application of Nanotechnology in Orthodontic Materials: A State-of-the-Art Review. Dent J (Basel). 2020;8(4).
34. Dean E, Söderlund A. What is the role of lifestyle behaviour change associated with non-communicable disease risk in managing musculoskeletal health conditions with special reference to chronic pain? BMC Musculoskelet Disord. 2015;16:87.
35. Delgado J, Bowman K, Clare L. Potentially inappropriate prescribing in dementia: a state-of-the-art review since 2007. BMJ open. 2020;10(1):e029172-e.
36. Dockrell L, Buggy DJ. The role of regional anaesthesia in the emerging subspecialty of onco-anaesthesia: a state-of-the-art review. Anaesthesia. 2021;76 Suppl 1:148-59.
37. Doucet J, Kiri L, O'Connell K, Kehoe S, Lewandowski RJ, Liu DM, et al. Advances in Degradable Embolic Microspheres: A State of the Art Review. Journal of functional biomaterials. 2018;9(1):14.
38. Ekman N, Taft C, Moons P, Mäkitalo Å, Boström E, Fors A. A state-of-the-art review of direct observation tools for assessing competency in person-centred care. Int J Nurs Stud. 2020;109:103634.
39. Elhegazy H, Eid MMM. A state-of-the-art-review on grey water management: a survey from 2000 to 2020s. Water Sci Technol. 2020;82(12):2786-97.
40. Evans DC, Wojda TR, Jones CD, Otey AJ, Stawicki SP. Intentional ingestions of foreign objects among prisoners: A review. World J Gastrointest Endosc. 2015;7(3):162-8.
41. Fede C, Gaudreault N, Fan C, Macchi V, De Caro R, Stecco C. Morphometric and dynamic measurements of muscular fascia in healthy individuals using ultrasound imaging: a summary of the discrepancies and gaps in the current literature. Surg Radiol Anat. 2018;40(12):1329-41.
42. Fernández-Llamazares Á, Garteizgogeascoa M, Basu N, Brondizio ES, Cabeza M, Martínez-Alier J, et al. A State-of-the-Art Review of Indigenous Peoples and Environmental Pollution. Integr Environ Assess Manag. 2020;16(3):324-41.
43. Frank GKW, Shott ME, DeGuzman MC. The Neurobiology of Eating Disorders. Child Adolesc Psychiatr Clin N Am. 2019;28(4):629-40.
44. Frost R, Rait G, Wheatley A, Wilcock J, Robinson L, Harrison Dening K, et al. What works in managing complex conditions in older people in primary and community care? A state-of-the-art review. Health Soc Care Community. 2020;28(6):1915-27.
45. Gavriilaki M, Kimiskidis VK, Gavriilaki E. Precision Medicine in Neurology: The Inspirational Paradigm of Complement Therapeutics. Pharmaceuticals (Basel). 2020;13(11).
46. Ghose S, Holloway L, Lim K, Chan P, Veera J, Vinod SK, et al. A review of segmentation and deformable registration methods applied to adaptive cervical cancer radiation therapy treatment planning. Artif Intell Med. 2015;64(2):75-87.
47. Hansen MM, Jones R, Tocchini K. Shinrin-Yoku (Forest Bathing) and Nature Therapy: A State-of-the-Art Review. International journal of environmental research and public health. 2017;14(8):851.
48. Hardy J, Haywood A, Rickett K, Sallnow L, Good P. Practice review: Evidence-based quality use of corticosteroids in the palliative care of patients with advanced cancer. Palliat Med. 2021;35(3):461-72.
49. Harmon KG, Drezner JA, Wilson MG, Sharma S. Incidence of sudden cardiac death in athletes: a state-of-the-art review. Heart. 2014;100(16):1227-34.
50. Harrill WC, Melon DE. A field guide to U.S. healthcare reform: The evolution to value-based healthcare. Laryngoscope Investig Otolaryngol. 2021;6(3):590-9.
51. Hasan B, Hansmann G, Budts W, Heath A, Hoodbhoy Z, Jing ZC, et al. Challenges and Special Aspects of Pulmonary Hypertension in Middle- to Low-Income Regions: JACC State-of-the-Art Review. J Am Coll Cardiol. 2020;75(19):2463-77.
52. Heiss WD, Rosenberg GA, Thiel A, Berlot R, de Reuck J. Neuroimaging in vascular cognitive impairment: a state-of-the-art review. BMC Med. 2016;14(1):174.
53. Higgins TS, Wu AW, Illing EA, Sokoloski KJ, Weaver BA, Anthony BP, et al. Intranasal Antiviral Drug Delivery and Coronavirus Disease 2019 (COVID-19): A State of the Art Review. Otolaryngol Head Neck Surg. 2020;163(4):682-94.
54. Hirschman KB, Hodgson NA. Evidence-Based Interventions for Transitions in Care for Individuals Living With Dementia. Gerontologist. 2018;58(suppl_1):S129-s40.
55. Hofkens PJ, Verrijcken A, Merveille K, Neirynck S, Van Regenmortel N, De Laet I, et al. Common pitfalls and tips and tricks to get the most out of your transpulmonary thermodilution device: results of a survey and state-of-the-art review. Anaesthesiol Intensive Ther. 2015;47(2):89-116.
56. Hsueh WD, Hwang PH, Abuzeid WM. Perioperative Management of Antithrombotic Therapy in Common Otolaryngologic Surgical Procedures: State of the Art Review. Otolaryngol Head Neck Surg. 2015;153(4):493-503.
57. Hull JH, Ansley L, Price OJ, Dickinson JW, Bonini M. Eucapnic Voluntary Hyperpnea: Gold Standard for Diagnosing Exercise-Induced Bronchoconstriction in Athletes? Sports Med. 2016;46(8):1083-93.
58. Hulteen RM, Waldhauser KJ, Beauchamp MR. Promoting Health-Enhancing Physical Activity: a State-of-the-art Review of Peer-Delivered Interventions. Current obesity reports. 2019;8(4):341-53.
59. Jahandideh-Tehrani M, Bozorg-Haddad O, Loáiciga HA. Application of non-animal-inspired evolutionary algorithms to reservoir operation: an overview. Environ Monit Assess. 2019;191(7):439.
60. Jawhari B, Ludwick D, Keenan L, Zakus D, Hayward R. Benefits and challenges of EMR implementations in low resource settings: a state-of-the-art review. BMC medical informatics and decision making. 2016;16(1):116-.
61. Johnson BJ, Choby GW, O'Brien EK. Chronic rhinosinusitis in patients with cystic fibrosis-Current management and new treatments. Laryngoscope Investig Otolaryngol. 2020;5(3):368-74.
62. Johnson M, Chen Y, Hovet S, Xu S, Wood B, Ren H, et al. Fabricating biomedical origami: a state-of-the-art review. International journal of computer assisted radiology and surgery. 2017;12(11):2023-32.
63. Jones EA, Shuman AG, Egleston BL, Liu JC. Common Pitfalls of Head and Neck Research Using Cancer Registries. Otolaryngol Head Neck Surg. 2019;161(2):245-50.
64. Kadado AJ, Islam A. Iatrogenic atrial septal defect following the MitraClip procedure: A state-of-the-art review. Catheter Cardiovasc Interv. 2021;97(7):E1043-e52.
65. Kadah A, Khoury T, Mari A, Mahamid M, Sbeit W. Lumen-apposing metal stents in interventional endoscopy: a state-of-the-art review with focus on technical and clinical successes and complications. Eur J Gastroenterol Hepatol. 2020;32(1):1-9.
66. Keshwani N, McLean L. State of the art review: Intravaginal probes for recording electromyography from the pelvic floor muscles. Neurourol Urodyn. 2015;34(2):104-12.
67. Ketelaar M, Bogossian A, Saini M, Visser-Meily A, Lach L. Assessment of the family environment in pediatric neurodisability: a state-of-the-art review. Dev Med Child Neurol. 2017;59(3):259-69.
68. Khoury T, Mizrahi M, Mahamid M, Daher S, Nadella D, Hazou W, et al. State of the art review with literature summary on gastric peroral endoscopic pyloromyotomy for gastroparesis. J Gastroenterol Hepatol. 2018;33(11):1829-33.
69. Kim C, Han J, Wu T, Bachert C, Fokkens W, Hellings P, et al. Role of Biologics in Chronic Rhinosinusitis With Nasal Polyposis: State of the Art Review. Otolaryngol Head Neck Surg. 2021;164(1):57-66.
70. Krutmann J, Liu W, Li L, Pan X, Crawford M, Sore G, et al. Pollution and skin: from epidemiological and mechanistic studies to clinical implications. J Dermatol Sci. 2014;76(3):163-8.
71. Kyd JM, Hotomi M, Kono M, Kurabi A, Pichichero M, Ryan A, et al. Panel 5: Immunology. Otolaryngol Head Neck Surg. 2017;156(4_suppl):S63-s75.
72. Lanzillotti AI, Sarudiansky M, Lombardi NR, Korman GP, L DA. Updated Review on the Diagnosis and Primary Management of Psychogenic Nonepileptic Seizure Disorders. Neuropsychiatr Dis Treat. 2021;17:1825-38.
73. Lechien JR, Akst LM, Hamdan AL, Schindler A, Karkos PD, Barillari MR, et al. Evaluation and Management of Laryngopharyngeal Reflux Disease: State of the Art Review. Otolaryngol Head Neck Surg. 2019;160(5):762-82.
74. Lee JJJ, Loh WP. A state-of-the-art review on badminton lunge attributes. Computers in biology and medicine. 2019;108:213-22.
75. Lehmann AE, Scangas GA, Bergmark RW, El Rassi E, Stankovic KM, Metson R. Periostin and Inflammatory Disease: Implications for Chronic Rhinosinusitis. Otolaryngol Head Neck Surg. 2019;160(6):965-73.
76. Liu B, Chi W, Li X, Li P, Liang W, Liu H, et al. Evolving the pulmonary nodules diagnosis from classical approaches to deep learning-aided decision support: three decades' development course and future prospect. J Cancer Res Clin Oncol. 2020;146(1):153-85.
77. Loerracher AK, Braunbeck T. Cytochrome P450-dependent biotransformation capacities in embryonic, juvenile and adult stages of zebrafish (Danio rerio)-a state-of-the-art review. Arch Toxicol. 2021;95(7):2299-334.
78. Loewen I, Jeffery CC, Rieger J, Constantinescu G. Prehabilitation in head and neck cancer patients: a literature review. J Otolaryngol Head Neck Surg. 2021;50(1):2.
79. Loo J, Spittle DA, Newnham M. COVID-19, immunothrombosis and venous thromboembolism: biological mechanisms. Thorax. 2021;76(4):412-20.
80. Luetke A, Meyers PA, Lewis I, Juergens H. Osteosarcoma treatment - where do we stand? A state of the art review. Cancer treatment reviews. 2014;40(4):523-32.
81. Maleitzke T, Elazaly H, Festbaum C, Eder C, Karczewski D, Perka C, et al. Mesenchymal Stromal Cell-Based Therapy-An Alternative to Arthroplasty for the Treatment of Osteoarthritis? A State of the Art Review of Clinical Trials. J Clin Med. 2020;9(7).
82. McCluskey SP, Plisson C, Rabiner EA, Howes O. Advances in CNS PET: the state-of-the-art for new imaging targets for pathophysiology and drug development. Eur J Nucl Med Mol Imaging. 2020;47(2):451-89.
83. McGarvey L, Rubin BK, Ebihara S, Hegland K, Rivet A, Irwin RS, et al. Global Physiology and Pathophysiology of Cough: Part 2. Demographic and Clinical Considerations: CHEST Expert Panel Report. Chest. 2021.
84. McKercher GR, Salmond JA, Vanos JK. Characteristics and applications of small, portable gaseous air pollution monitors. Environ Pollut. 2017;223:102-10.
85. Megna M, Napolitano M, Costa C, Balato N, Patruno C. Waste exposure and skin diseases. G Ital Dermatol Venereol. 2017;152(4):379-82.
86. Meima-van Praag EM, Buskens CJ, Hompes R, Bemelman WA. Surgical management of Crohn's disease: a state of the art review. Int J Colorectal Dis. 2021;36(6):1133-45.
87. Meister KD, Pandian V, Hillel AT, Walsh BK, Brodsky MB, Balakrishnan K, et al. Multidisciplinary Safety Recommendations After Tracheostomy During COVID-19 Pandemic: State of the Art Review. Otolaryngol Head Neck Surg. 2021;164(5):984-1000.
88. Mennuni S, Rubattu S, Pierelli G, Tocci G, Fofi C, Volpe M. Hypertension and kidneys: unraveling complex molecular mechanisms underlying hypertensive renal damage. J Hum Hypertens. 2014;28(2):74-9.
89. Monti M, Vertogen B, Masini C, Donati C, Lilli C, Zingaretti C, et al. Hydroxychloroquine as Prophylaxis for COVID-19: A Review. Front Pharmacol. 2020;11:605185.
90. Moore J, Castellanos S, Xu S, Wood B, Ren H, Tse ZTH. Applications of Wireless Power Transfer in Medicine: State-of-the-Art Reviews. Ann Biomed Eng. 2019;47(1):22-38.
91. Morin DP, Bernard ML, Madias C, Rogers PA, Thihalolipavan S, Estes NA, 3rd. The State of the Art: Atrial Fibrillation Epidemiology, Prevention, and Treatment. Mayo Clin Proc. 2016;91(12):1778-810.
92. Moro E, Degli Esposti E, Borghese G, Manzara F, Zanello M, Raimondo D, et al. The Impact of Hormonal Replacement Treatment in Postmenopausal Women with Uterine Fibroids: A State-of-the-Art Review of the Literature. Medicina (Kaunas, Lithuania). 2019;55(9):549.
93. Nascimento L, Bonfati LV, Freitas MB, Mendes Junior JJA, Siqueira HV, Stevan SL, Jr. Sensors and Systems for Physical Rehabilitation and Health Monitoring-A Review. Sensors (Basel). 2020;20(15).
94. Nogay HS, Adeli H. Machine learning (ML) for the diagnosis of autism spectrum disorder (ASD) using brain imaging. Rev Neurosci. 2020.
95. Nowinski WL, Walecki J, Półtorak-Szymczak G, Sklinda K, Mruk B. Ischemic infarct detection, localization, and segmentation in noncontrast CT human brain scans: review of automated methods. PeerJ. 2020;8:e10444.
96. Oliveira NL, Ribeiro F, Alves AJ, Campos L, Oliveira J. The effects of exercise training on arterial stiffness in coronary artery disease patients: a state-of-the-art review. Clin Physiol Funct Imaging. 2014;34(4):254-62.
97. Onyura B, Crann S, Freeman R, Whittaker MK, Tannenbaum D. The state-of-play in physician health systems leadership research. Leadersh Health Serv (Bradf Engl). 2019;32(4):620-43.
98. Park JC, Altman KW, Prasad VMN, Broadhurst M, Akst LM. Laryngeal Leukoplakia: State of the Art Review. Otolaryngol Head Neck Surg. 2021;164(6):1153-9.
99. Pecchia L, Castaldo R, Montesinos L, Melillo P. Are ultra-short heart rate variability features good surrogates of short-term ones? State-of-the-art review and recommendations. Healthcare technology letters. 2018;5(3):94-100.
100. Petrovsky DV, McPhillips MV, Li J, Brody A, Caffeé L, Hodgson NA. Sleep disruption and quality of life in persons with dementia: A state-of-the-art review. Geriatric nursing (New York, NY). 2018;39(6):640-5.
101. Piette JD, List J, Rana GK, Townsend W, Striplin D, Heisler M. Mobile Health Devices as Tools for Worldwide Cardiovascular Risk Reduction and Disease Management. Circulation. 2015;132(21):2012-27.
102. Ramakreshnan L, Aghamohammadi N, Fong CS, Sulaiman NM. A comprehensive bibliometrics of 'walkability' research landscape: visualization of the scientific progress and future prospects. Environ Sci Pollut Res Int. 2021;28(2):1357-69.
103. Rameau A, Hong RS, Djalilian H, Erbele ID, Phillips KM, Capasso R, et al. New Medical Device and Therapeutic Approvals in Otolaryngology: State of the Art Review of 2019. OTO Open. 2020;4(2):2473974x20932506.
104. Raoufi E, Hemmati M, Eftekhari S, Khaksaran K, Mahmodi Z, Farajollahi MM, et al. Epitope Prediction by Novel Immunoinformatics Approach: A State-of-the-art Review. Int J Pept Res Ther. 2020;26(2):1155-63.
105. Rice D, McNair P, Huysmans E, Letzen J, Finan P. Best Evidence Rehabilitation for Chronic Pain Part 5: Osteoarthritis. J Clin Med. 2019;8(11).
106. Rizzo A, Tondera K, Pálfy TG, Dittmer U, Meyer D, Schreiber C, et al. Constructed wetlands for combined sewer overflow treatment: A state-of-the-art review. Sci Total Environ. 2020;727:138618.
107. Rosenfeld RM. Tympanostomy Tube Controversies and Issues: State-of-the-Art Review. Ear Nose Throat J. 2020;99(1_suppl):15s-21s.
108. Sabet Sarvestani A, Sienko KH. Medical device landscape for communicable and noncommunicable diseases in low-income countries. Global Health. 2018;14(1):65.
109. Sbeit W, Kadah A, Mahamid M, Pellicano R, Mari A, Khoury T. A State-of-the-Art Review on the Evolving Utility of Endoscopic Ultrasound in Liver Diseases Diagnosis. Diagnostics (Basel). 2020;10(8).
110. Scotto G, Fazio V, Lo Muzio L. Tuberculosis in the immigrant population in Italy: state-of-the-art review. Infez Med. 2017;25(3):199-209.
111. Shao S, Hua Y, Yang Y, Liu X, Fan J, Zhang A, et al. Salt reduction in China: a state-of-the-art review. Risk management and healthcare policy. 2017;10:17-28.
112. Shojaee S, Roy-Chowdhuri S, Safi J, Grosu HB. Cytologic Investigations for the Diagnosis of Malignant Pleural Effusion in Non-small Cell Lung Cancer: State-of-the-art Review for Pulmonologists. J Bronchology Interv Pulmonol. 2021.
113. Singh AD, Singal AK, Mian A, Kapadia SR, Hedrick DP, Kanaa NA, et al. Recurrent Drug-Eluting Stent In-Stent Restenosis: A State-of-the-Art Review of Pathophysiology, Diagnosis, and Management. Cardiovasc Revasc Med. 2020;21(9):1157-63.
114. Sohlberg MM, MacDonald S, Byom L, Iwashita H, Lemoncello R, Meulenbroek P, et al. Social communication following traumatic brain injury part I: State-of-the-art review of assessment tools. Int J Speech Lang Pathol. 2019;21(2):115-27.
115. Stiles-Shields C, Ho J, Mohr DC. A review of design characteristics of cognitive behavioral therapy-informed behavioral intervention technologies for youth with depression and anxiety. Digit Health. 2016;2:2055207616675706.
116. Stropahl M, Besser J, Launer S. Auditory Training Supports Auditory Rehabilitation: A State-of-the-Art Review. Ear Hear. 2020;41(4):697-704.
117. Suhaimi NS, Mountstephens J, Teo J. EEG-Based Emotion Recognition: A State-of-the-Art Review of Current Trends and Opportunities. Comput Intell Neurosci. 2020;2020:8875426.
118. Sutton A, Clowes M, Preston L, Booth A. Meeting the review family: exploring review types and associated information retrieval requirements. Health Info Libr J. 2019;36(3):202-22.
119. Szerman N, Peris L. Precision Psychiatry and Dual Disorders. J Dual Diagn. 2018;14(4):237-46.
120. Thoreau R. The impact of mobility scooters on their users. Does their usage help or hinder?: A state of the art review. Journal of transport & health. 2015;2(2):269-75.
121. Thorne T, Olson K, Wismer W. A state-of-the-art review of the management and treatment of taste and smell alterations in adult oncology patients. Supportive care in cancer : official journal of the Multinational Association of Supportive Care in Cancer. 2015;23(9):2843-51.
122. Tingi E, Syed AA, Kyriacou A, Mastorakos G, Kyriacou A. Benign thyroid disease in pregnancy: A state of the art review. Journal of clinical & translational endocrinology. 2016;6:37-49.
123. Tolo VT, Herring JA. Scoliosis-specific exercises: A state of the Art Review. Spine Deform. 2020;8(2):149-55.
124. Tsang G, Xie X, Zhou SM. Harnessing the Power of Machine Learning in Dementia Informatics Research: Issues, Opportunities, and Challenges. IEEE Rev Biomed Eng. 2020;13:113-29.
125. Ubando AT, Africa ADM, Maniquiz-Redillas MC, Culaba AB, Chen WH. Reduction of particulate matter and volatile organic compounds in biorefineries: A state-of-the-art review. J Hazard Mater. 2021;403:123955.
126. van der Kruk E, Reijne MM. Accuracy of human motion capture systems for sport applications; state-of-the-art review. European journal of sport science. 2018;18(6):806-19.
127. Veerasamy M, Bagnall A, Neely D, Allen J, Sinclair H, Kunadian V. Endothelial dysfunction and coronary artery disease: a state of the art review. Cardiology in review. 2015;23(3):119-29.
128. Venturella R, Quaresima P, Micieli M, Rania E, Palumbo A, Visconti F, et al. Non-obstetrical indications for cesarean section: a state-of-the-art review. Archives of gynecology and obstetrics. 2018;298(1):9-16.
129. Vervoort D, Swain JD, Pezzella AT, Kpodonu J. Cardiac Surgery in Low- and Middle-Income Countries: A State-of-the-Art Review. Ann Thorac Surg. 2021;111(4):1394-400.
130. Violanti JM, Charles LE, McCanlies E, Hartley TA, Baughman P, Andrew ME, et al. Police stressors and health: a state-of-the-art review. Policing (Bradford, England). 2017;40(4):642-56.
131. von Allmen DC, Francey LJ, Rogers GM, Ruben MD, Cohen AP, Wu G, et al. Circadian Dysregulation: The Next Frontier in Obstructive Sleep Apnea Research. Otolaryngol Head Neck Surg. 2018;159(6):948-55.
132. Waked K, Schepens M. State-of the-art review on the renal and visceral protection during open thoracoabdominal aortic aneurysm repair. Journal of visualized surgery. 2018;4:31-.
133. Warren MF, Livingston KA. Implications of Vitamin D Research in Chickens can Advance Human Nutrition and Perspectives for the Future. Curr Dev Nutr. 2021;5(5):nzab018.
134. You E, Lin V, Mijovic T, Eskander A, Crowson MG. Artificial Intelligence Applications in Otology: A State of the Art Review. Otolaryngol Head Neck Surg. 2020;163(6):1123-33.
135. Zallmann M, Leventer RJ, Mackay MT, Ditchfield M, Bekhor PS, Su JC. Screening for Sturge-Weber syndrome: A state-of-the-art review. Pediatric dermatology. 2018;35(1):30-42.
136. Zanello M, Borghese G, Manzara F, Degli Esposti E, Moro E, Raimondo D, et al. Hormonal Replacement Therapy in Menopausal Women with History of Endometriosis: A Review of Literature. Medicina (Kaunas). 2019;55(8).
137. Zhang T, Chen J, Li F, Zhang K, Lv H, He S, et al. Intelligent fault diagnosis of machines with small & imbalanced data: A state-of-the-art review and possible extensions. ISA Trans. 2021.
138. Zhao Z, Jordan S, Tse ZTH. Devices for image-guided lung interventions: State-of-the-art review. Proc Inst Mech Eng H. 2019;233(4):444-63.
139. Zhao Z, Poyhonen J, Chen Cai X, Sophie Woodley Hooper F, Ma Y, Hu Y, et al. Augmented reality technology in image-guided therapy: State-of-the-art review. Proc Inst Mech Eng H. 2021:9544119211034357.
